# A Computational Method Based on the Integration of Heterogeneous Networks for Predicting Disease-Gene Associations

**DOI:** 10.1371/journal.pone.0024171

**Published:** 2011-09-02

**Authors:** Xingli Guo, Lin Gao, Chunshui Wei, Xiaofei Yang, Yi Zhao, Anguo Dong

**Affiliations:** 1 School of Computer Science and Technology, Xidian University, Xi'an City, Shaanxi Province, People's Republic of China; 2 School of Software Engineering, Xidian University, Xi'an City, Shaanxi Province, People's Republic of China; 3 Institute of Computing Technology, Chinese Academy of Sciences, Beijing, People's Republic of China; 4 School of Science, Chang'an University, Xi'an City, Shaanxi Province, People's Republic of China; University of Vermont, United States of America

## Abstract

The identification of disease-causing genes is a fundamental challenge in human health and of great importance in improving medical care, and provides a better understanding of gene functions. Recent computational approaches based on the interactions among human proteins and disease similarities have shown their power in tackling the issue. In this paper, a novel systematic and global method that integrates two heterogeneous networks for prioritizing candidate disease-causing genes is provided, based on the observation that genes causing the same or similar diseases tend to lie close to one another in a network of protein-protein interactions. In this method, the association score function between a query disease and a candidate gene is defined as the weighted sum of all the association scores between similar diseases and neighbouring genes. Moreover, the topological correlation of these two heterogeneous networks can be incorporated into the definition of the score function, and finally an iterative algorithm is designed for this issue. This method was tested with 10-fold cross-validation on all 1,126 diseases that have at least a known causal gene, and it ranked the correct gene as one of the top ten in 622 of all the 1,428 cases, significantly outperforming a state-of-the-art method called PRINCE. The results brought about by this method were applied to study three multi-factorial disorders: breast cancer, Alzheimer disease and diabetes mellitus type 2, and some suggestions of novel causal genes and candidate disease-causing subnetworks were provided for further investigation.

## Introduction

Computational investigation of gene functions in the context of complex biological systems is promoted greatly by the accumulation of high-throughput data, of which protein-protein interaction data have been exploited to identify disease-causing genes, based on the observation that genes implicated in a specific or similar diseases tend to be located in a specific neighbourhood in the protein-protein interaction network [1], [2], [3]. The identification of genes involved in a specific disease has long been a challenge in the study of human genetics. In addition to traditional gene-mapping approaches, many computational methods based on gene functions have appeared, which was reviewed by Oti and Brunner in [3]. Recently, a few computational approaches for candidate gene prioritization have been developed which exploit both the protein-protein interactions and the disease phenotypic similarities. Lage et al. [4] scored a candidate protein based on the involvement of its direct network neighbours involved in a similar disease, in which a new disease similarity measure was also given and applied for prioritizing both the protein complex and the candidate disease gene in the protein complex. Kohler et al. [5] presented a method for prioritization of candidate genes by use of a global network distance measure-random walk analysis-for definition of similarities in the protein-protein interaction network. Wu et al. [6] proposed a computational framework that integrates human protein-protein interactions, phenotype similarities, and known gene-phenotype associations to capture the complex relationships between disease phenotypes and genotypes. They defined the global concordance score between the phenotype similarity profile and the gene closeness profile as the disease-gene association score. Furthermore, a tool named CIPHER was developed to predict and prioritize candidate disease-causing genes. In their follow-up work [7], they studied the consistency between the disease phenotypic overlap and genetic overlap via the network alignment technique systematically and quantitatively. Vanunu et al. introduced PRINCE [8], a global method for prioritizing candidate genes that simulates a process where proteins for which prior information exists pump information to their neighbours in the protein-protein interaction network. In PRINCE, for a given disease the prioritization is done iteratively over the entire protein interaction network, and each protein propagates the information received in the previous iteration to its neighbours.

**Figure 1:**
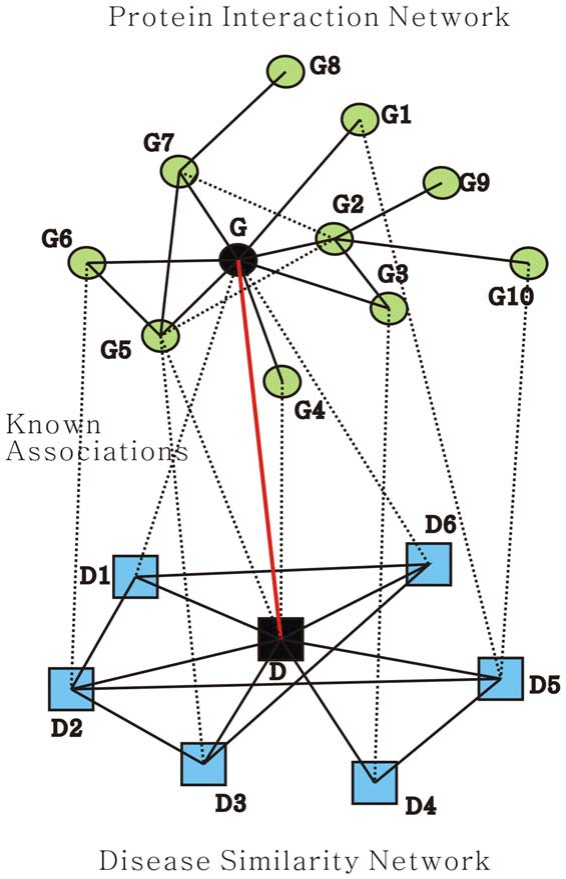
Principle of our method. In Figure 1, green round nodes represent proteins, and blue square nodes represent diseases, with each dot line connecting a green round node and a blue square node indicating a known disease-gene association. The red line connecting gene 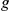 and disease 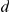 is a candidate disease-gene association remaining to be estimated, which is measured by the associations indicated by black dot line in this figure iteratively in our method.

Although many approaches have been developed for prioritizing candidate disease-causing genes based on exploiting the protein-protein interaction network and phenotype similarities, most of which deal with the disease-gene association score based on the association between the diseases similar to the query disease and their involved genes independently. In this work, the modular nature of the genetic diseases [3], [9] and the consistency between the disease phenotypic overlap [10] and genetic overlap [11] are fully exploited. For this purpose, the disease similarity network and the protein-protein interaction network are incorporated systematically and comprehensively in a simple and compact manner to formulate the computation of the prioritization scores. As for a single disease gene association score function, both the similar diseases in the disease similarity network and neighbouring genes in the protein-protein interaction network are considered because of the modular nature of the genetic diseases. What is more, all the association scores between the similar diseases and neighbouring genes would be integrated into the iterative computation of this single disease gene association score. This is illustrated in Figure 1.

Our method is used to analyse disease-gene association data from the Online Mendelian Inheritance in Man (OMIM) [12] and to test, in the 10-fold cross-validation setting, the utility in prioritizing genes for all the diseases with at least one known gene. The performance of our method is evaluated in comparison to the method PRINCE. Results show that our method outperforms PRINCE significantly in the gene prioritization task. Our method is also applied to the study of three multi-factorial diseases-Breast Cancer, Alzheimer Disease and Diabetes Mellitus Type 2, for which some novel causal genes and related disease subnetworks are suggested.

This paper is structured as follows: In Section “Results”, a comparison of our method and PRINCE is first made with 10-fold cross-validation. Then our method is further validated on the three types of control data set, with its robustness also evaluated. Finally, we perform a case study on three multi-factorial diseases-Breast Cancer, Alzheimer Disease and Diabetes Mellitus Type 2. In Section “Discussions and Conclusions”, the success and improvements of this method are described, with further applications of this method also discussed and prospected. In Section “Methods” we introduce the principle of this method, the network construction, and the iterative algorithm for the computation of the disease gene association score.

## Results

### Materials and Implementation

The 1428 known disease-gene associations and the protein-protein interactions used to create the disease gene association matrix 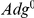 and the protein-protein interaction network 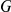, respectively, are downloaded from Cipher's website [13]. According to the declaration in Cipher [6], the disease-gene associations are from the OMIM knowledge database [12], and the protein-protein interactions from the Human Protein Reference Database(HPRD) [14]. The disease similarity data constructed by van Driel et al. [10] are downloaded from MimMiner's website [15]. All these data will be illustrated carefully later in the Section “Methods”.

In this method, there are three parameters to be tuned: (1) the threshold parameter 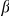 which is used to filter out the disease similarity and the prior association score smaller than it, and is set as “0.5”; (2) 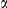, which controls the relative importance of the prior information in the computation of the disease-gene association scores. We choose “0.6” for it and the other values above “0.6” can not improve the performance of the method; and (3) the number of the iterations. 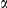 and 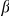 are tuned by the performance of the algorithm in the cross-validation tests. The iterative computation will stop by the mean square deviation of the disease-gene association score matrix between the 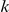 th iteration and the 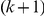 th iteration. Once the mean square deviation is not greater than 0.00001, the algorithm will be stopped.

Our method has been implemented in MATLAB, and PRINCE has been reimplemented on our input data sets. Their parameters were also tuned in the cross-validation test, in which the parameter 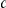 and 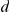 were tuned as −1414 and 528, respectively, to get the best performance, and the relative importance of the prior knowledge and the mean square deviation were set as the same as those in our method.

All the computational experiments were executed on four cores of Intel(R) Xeon(R) CPU E5504 @2.00Ghz.The MATLAB code and data sets described herein are available upon request.

### Precision, recall and irrelevant control set

According to the standard definitions of precision and recall in Formula (1), which were given by Lage et al. [4].
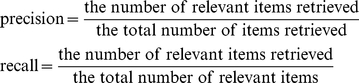
(1)


For a given-rank threshold, precision is the fraction of the relevant genes retrieved among all the genes retrieved (the number of relevant genes retrieved/the number of genes retrieved) and recall is the proportion of the relevant genes retrieved to all the relevant genes in collection (the number of relevant genes retrieved/the number of relevant genes). Here, the relevant genes are considered as the known disease genes for each disease, and the total number of items (genes) retrieved is the total number of genes above the rank threshold.

To compute the precision and recall, the relevant items and irrelevant items should be constructed. First and foremost, the known disease-gene associations are relevant items here. As for the irrelevant items, we associated the genes that are not known to any disease with a disease artificially, and these disease-gene associations are considered as irrelevant items and constitute the irrelevant control set. It should be known that the genes, which are not associated with any disease in our disease-gene association set and called “unassociated genes”, are not “irrelevant” and just “unknown to us”. In our method, three types of irrelevant control set are constructed. One is the whole genome wide control set, another is the random control set and the last one is the artificial linkage interval control set. As for the random control set in 10-fold cross-validation, we divided all the 1428 disease-gene associations into ten subdivisions averagely, with about 142 diseases and 142 disease-gene associations in each subdivision. For each subdivision, we randomly selected 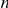 genes from the set of “unassociated genes”. For each disease involved in the subdivision, we constructed 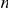 disease-gene associations with the 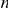 random selected genes. So, there are about 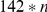 disease-gene associations constructed artificially, which constitute the random control set and are considered as irrelevant items. There are about 142 known disease-gene associations, which are considered as relevant items. Both the irrelevant and relevant items are measured by their ranks in the whole genome to compute precision and recall. All ten subdivisions are done separately in the same way as above. For a given rank 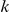, the final precision and recall are the average results of all ten subdivisions. As for the whole genome wide control set, all the “unassociated genes” in the protein interaction network rather than random selected 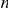 genes are used, and the irrelevant items and the irrelevant control set are constructed in the same way as above. As regard to simulating the real-life situation in which one or more susceptible linkage intervals rather than specific genes have been associated with some disease, an artificial linkage interval around a known disease-causing gene is constructed according to the genes' coordinates on the whole genome, and this is motivated by the method used in Lage et al. [4]. We extracted no more than 100 genes around the known disease gene on the same chromosome, and these genes are used to construct the irrelevant items and the irrelevant control set as above. The tests were performed on the three irrelevant control sets, and the results will be described in detail later.

### A comparison between our method and the method PRINCE

Only a comparison was made between our method and the state-of-the-art method PRINCE because in PRINCE both the random-walk based method of [5] and the Cipher method [6] were reimplemented and evaluated on the same input data. We reimplemented the method PRINCE on our input data and made the comparison with it. Our method was compared with PRINCE by the 10-fold cross-validation procedure. In each test of 10-fold cross-validation, 1/10 of the known associations in the disease-gene association set were removed, each method being evaluated by its success in recovering the hidden association. For a given-rank threshold 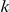, if the hidden disease-gene association was ranked within the top 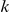 over the entire protein interaction network, it could be said that the association was successfully recovered. The two methods were evaluated in performance in terms of precision versus recall when varying the rank threshold 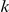.

**Figure 2:**
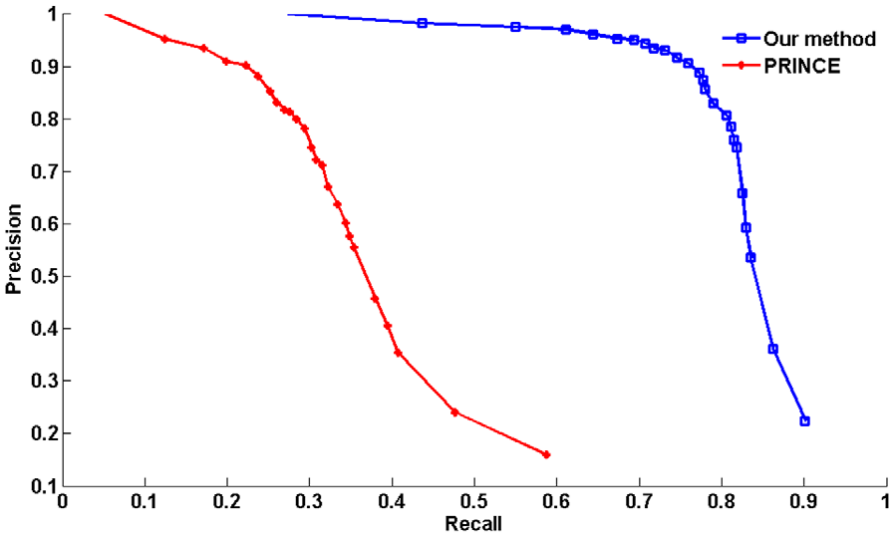
Comparison in performance between our method and PRINCE. A comparison between our method and PRINCE. We can see that our method gives a high precision as well as a fairly high recall, and this is superior to that from PRINCE.

**Figure 3:**
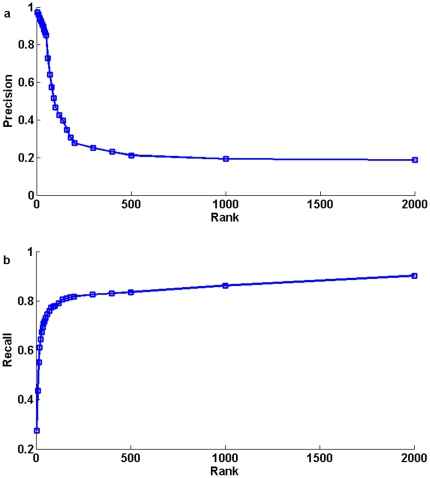
A plot of precision and recall versus threshold 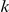 of our method. Figure 3-a A plot of precision versus threshold 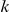. Figure 3-b A plot of recall versus threshold 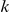, where 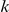 means that the gene was ranked within top 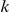 in our method.

The results obtained by prioritizing candidates on all 1126 diseases in the 10-fold cross-validation show that our method is superior to PRINCE in both precision and recall (Figure 2). Of all 1126 diseases, in terms of 10-fold cross-validation on the random control set of size 2000, there are 633 different predictions ranked within top 10, among which there are 622 correctly identified disease genes, so that the precision at this threshold is 98.3%. At the same threshold, the recall is 43.6%. A plot of precision versus threshold 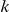 shows the proportionality between the rank and the chance that the candidate gene is the correctly identified disease gene. Candidates ranked within top 100 are correct in more than 85.7% of the cases (Figure 3-a). That is, top ranked candidates are very likely to be correct disease-causing genes. Another plot of recall versus rank threshold 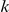 is depicted in Figure 3-b.

One type of failure to reconstruct the known disease gene association should be recognized. In our method, the topological correlation between the disease similarity network and the protein-protein interaction network are considered fairly in combination with the prior information on the disease-gene associations, and as a result, the well-connected genes in the protein-protein interaction network may tend to be top ranked.

### Tests on three irrelevant control sets

**Figure 4:**
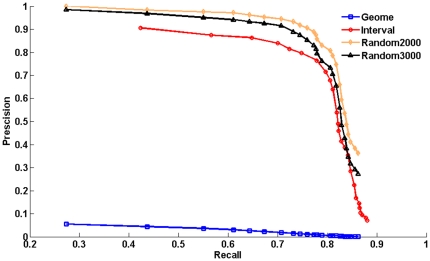
The performance of our method for three types of control data set. The method was tested on the whole genome, random control set and simulated interval data set.

Our method was tested on three types of irrelevant control set: the whole genome wide control set, the random control set and the artificial linkage interval control set. For the random control set, we randomly selected two thousand or three thousand “unassociated genes” to construct the irrelevant control set and the irrelevant items. The two other irrelevant control sets and irrelevant items are constructed in the way as described above. The 10-fold cross-validation tests were performed and the result analysis was made on the three irrelevant control sets. The results on the three types of control set are shown in Figure 4. Ranking over the whole genome is of great importance because many OMIM phenotypes have no causative genes till now. In terms of the 10-fold cross-validation, our method is used to successfully rank the known disease-causing gene as one of the top five from the 8919 genes in the protein interaction network for 391 cases, with a precision of 0.0548 obtained. It is natural that the performance of our method for the whole genome control set should be inferior to that for either the random control set or the simulated interval control set because the former control set is much larger than the other two. The method performed worse for the simulated interval set than for the random control set, which may be attributed to the incompleteness of protein-protein interaction data. In the construction of the artificial linkage interval control set, we chose the upstream and downstream 50 genes about the known disease gene on the same chromosome to simulate the linkage interval, and at the same time these 100 genes must be in the protein-protein interaction network. It can be expected that the linkage analysis in combination with the system approaches based on the biological interaction networks would give more powerful insights in identifying novel disease-causing genes.

### Robustness of Our Method

We tested our method, biased towards the disease similarity information and the protein-protein interaction networks. We randomly selected 1/5 the disease gene associations from our data set and 1000 genes from the protein-protein interaction network, and then added white Gaussian noise on 1/5 the disease similarity data and the interactions among all the 1000 genes to test the robustness of our method. In terms of the 10-fold cross-validation, the method can give a precision of 0.053 and 0.049, respectively, on the genome wide control set, and the precision degradation is small compared with the original precision of about 0.055. The results indicate that our method does not rely heavily on known disease similarity information or that it is not biased towards the better characterized genes heavily in the protein-protein interaction network.

### Case Study

**Figure 5:**
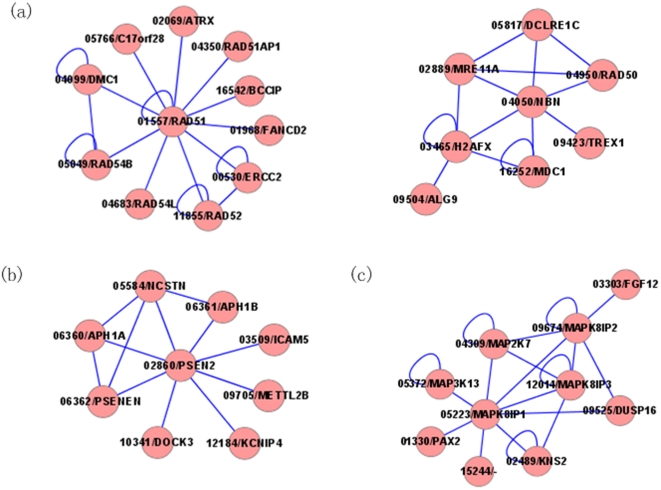
Subnetworks involved in disease for three cases. Four examples of putative protein subnetworks associated with the three cases are shown in Figure 5. The node in the figure represents the protein/gene, and the HPRD ID and gene symbol are given and separated by the slant ‘/’. The two putative disease subnetworks in Figure 5-a were discovered for Breast Cancer. The putative disease subnetwork in Figure 5-b was associated with Alzheimer Disease. The putative disease subnetwork in Figure 5-c was related to Diabetes Mellitus, Type 2.

To further demonstrate the power of our method, we proceeded to execute our method on multifactorial disorders. Breast Cancer (MIM:114800), Alzheimer Disease (MIM:104300), and Diabetes Mellitus Type 2 (MIM:125853) were selected for case studies. First, the ranks for the known disease genes or susceptibility factors implicated in the three cases are listed in Table 1. Second, we checked the top ranked candidate genes for these cases in the protein-protein interaction network, and at the same time a clustering algorithm called PageRankNibble [16] was performed over the protein-protein interaction network to discover the functional subnetworks. The clustering algorithm PageRankNibble is based on the random walk and PageRank vector. For a given starting protein in the protein-protein interaction network, a subnetwork near the starting protein may be found, and the computing time is proportional to the size of the subnetwork. Because of this property the algorithm PageRankNibble was used to discover the subnetworks (which are considered as functional modules and may be disease associated subnetworks) near all the known disease-causing genes. First we filter these subnetworks by their sizes and the ranks of the genes in them, and then a web server g:Profiler with default parameters [17], [18] was used to analyse these subnetworks. Some subnetworks are given as examples in Figure 5. The genes and their ranks in these subnetworks (Figure 5) are also listed in Table 2. The primary input to the web server g:Profiler is a list of gene, protein, or probe identifiers from any of the currently supported species [17]. Here in our analysis a list of genes in every subnetwork was provided as the input to g:Profiler. The typical output of g:Profiler is a set of enriched functional terms. Every term is accompanied by the size of the query and the term gene lists, their overlap and the statistical significance (P-value) of such enrichment [17]. In our analysis of the gene list in one subnetwork, we only focused on biological processes, molecular functions, subcellular localisations and pathways, and the number of genes annotated by the term and the P-value of term enrichment are analyzed and summarized later for each case study.

**Table 1:** The ranks of known disease-causing or susceptibility genes for three cases on the whole genome.

Breast cancer (MIM:114480)							
Gene	Rank	Gene	Rank	Gene	Rank	Gene	Rank
BRCA1	1	BRCA2	3	PIK3CA	6	NCOA3	261
BRIP1	5	RAD51	2	NBN	4	RAD51C	6369
TP53	10	RB1CC1	6223	AR	13	STK11	166
CHEK2	21	CDH1	407	PPM1D	4732	PTEN	7
CASP8	409	TGFB1	1881	NQO1	763	AKT1	28
HMMR	5789	ATM	11	BARD1	58		

Alzheimer Disease (MIM:104300)							
Gene	Rank	Gene	Rank	Gene	Rank	Gene	Rank
APP	1	PSEN2	2	ABCA2	7388	TF	1090
A2M	651	HFE	1267	LRP1	14	VEGF	2195
NOS3	809	TNF	2232	PSEN1	7		

Diabetes Mellitus, Type 2 (MIM:125853)							
Gene	Rank	Gene	Rank	Gene	Rank	Gene	Rank
SLC2A4	6	TCF2	5	MAPK8IP1	7	KCNJ15	7019
IRS1	2	PPP1R3A	8	NEUROD1	3	PPARG	1002
INSR	1	IPF1	4	KCNJ11	200	HNF4A	193
AKT2	117	ENPP1	158	IL6	4490	PTPN1	22
GPD2	2894	TCF1	9	TCF7L2	593		

In Table 1, both the known disease-causing genes and the susceptibility genes for three cases of Breast Cancer, Alzheimer Disease and Diabetes Mellitus Type 2 are listed, altogether with the corresponding rank in the whole genome.

**Table 2:** The ranks of genes in candidate disease subnetworks.

Genes in Fig. 5(a) and their ranks									
RAD52	112	DMC1	147	BCCIP	25	RAD54L	149	ERCC2	133
ATRX	145	C17orf28	144	RAD54B	121	RAD51AP1	148	RAD51	2
TREX1	108	RAD50	24	MRE11A	68	H2AFX	20	FANCD2	12
NBN	4	DCLRE1C	23	MDC1	17	ALG9	5758		
Genes in Fig. 5(b) and their ranks									
NCSTN	6	KCNIP4	104	APH1A	35	DOCK3	40	APH1B	36
ICAM5	28	PSENEN	37	PSEN2	2	METTL2B	39		
Genes in Fig. 5(c) and their ranks									
MAPK8IP3	97	MAP3K13	166	MAPK8IP2	98	MAP2K7	121	MAPK8IP1	7
DUSP16	120	FGF12	2464	KNS2	110	PAX2	162	15244/-	74

In Table 2, the genes in the candidate disease subnetworks (in Fig. 5) and their ranks are listed.

### Results for Breast Cancer

The section on the overview of Breast Cancer (MIM:114480) in OMIM gives a list of 23 susceptibility genes (January, 2011), which are also characterized by the protein-protein interaction network. The rank results of the genome-wide prioritization scores for the known disease and susceptibility genes are listed in Table 1. Our method assigned the No. 1 rank to the known disease genes in our data and also high ranks to most of the known breast cancer causative genes which are not in our data, with 15 of these 23 genes ranked in the top 300 of the ranked whole genome (Table 1), and with 300 being a reasonable number for the high-resolution single nucleotide polymorphism (SNP) association analysis of a complex disease in human population [19].

Next, we inferred Breast Cancer-related subnetworks by a clustering method PageRankNibble, with two of such subnetworks shown in Figure 5(a). The ranks of the genes in such subnetworks are also given in Table 2. We can see that the genes in such two subnetworks are ranked within top 200, except the gene ALG9 in the rightmost subnetwork of Figure 5(a). Also we examined the gene function in terms of GO [20] annotations and KEGG [21] pathway enrichment. This enrichment analysis is carried out on the g:Profiler web server. The leftmost subnetwork in Figure 5(a) contains 11 proteins, 6 of which are known to be involved in the M phase of the meiotic cell cycle (p-value = 1.08e-11), 7 of which are related to DNA recombination (p-value = 5.28e-14), 10 of which respond to the DNA damage stimulus (p-value = 7.27e-14), and 10 of which are associated with DNA repair (p-value = 7.17e-15). There are 8 proteins in the rightmost subnetwork, and the gene MDC1 is skipped because of ambiguous hits in GO database. 6 of 7 are related to the response to DNA damage stimulus (p-value = 8.67e-10), 6 of 7 are correlated to DNA recombination (p-value = 2.10e-13), 6 of 7 are involved in DNA repair (p-value = 1.86e-10), and 5 of 7 genes are connected with double-strand break repair (p-value = 7.55e-12). All this agrees well with the current knowledge on the breast cancer [22].

### Results for Alzheimer Disease

The section on the overview of Alzheimer(MIM:104300) in OMIM gives a list of 11 susceptibility genes (January, 2011), which are also characterized by the protein-protein interaction network. The rank results of the genome-wide prioritization scores for the known disease and susceptibility genes are listed in Table 1. The known disease genes in our data are top ranked, and high ranks are also given to most of the known Alzheimer causative genes which were not in our data, with 4 of these 11 genes ranked within the top 300 of the ranked whole genome (Table 1).

We made the same clustering analysis for Alzheimer Disease. We inferred Alzheimer related subnetworks, with one of such subnetworks shown in Figure 5(b). The ranks of the genes in this subnetwork are ranked within top 40, except for the gene KCNIP4. In the result analysis of the g:Profiler on the subnetwork in Figure 5(b), we can see that 5 of those genes are enriched in cell death and its regulation, that 5 of them are related to membrane protein intracellular domain proteolysis (p-value = 2.76e-15), that 5 of them are involved in induction of apoptosis (p-value = 2.23e-07), that 4 of them are correlated to Alzheimer's disease pathway (p-value = 4.15e-06), and that 4 of them are connected with Notch signaling pathway (p-value = 2.45e-08). Almost all the knowledge agrees well with the current knowledge on Alzheimer Disease [23], [24].

### Results for Diabetes Mellitus Type 2

The section on the overview of Diabetes Mellitus, Type 2 (MIM:125853) in OMIM gives a list of 20 susceptibility genes (January, 2011), which are characterized by the protein-protein interaction network. The rank results of the genome-wide prioritization scores for the known disease-causing genes and susceptibility genes are listed in Table 1. Our method assigned the top ranked to the known disease genes in our data and also high ranks to most of the known Diabetes Mellitus causative genes which were not in our data, with 15 of these 20 genes ranked within the top 300 of the ranked whole genome (Table 1).

The subnetworks related with Diabetes Mellitus, Type 2 were discovered by PageRankNibble, with one of such subnetworks shown in Figure 5(c). We ranked 9 of the genes in this subnetwork within top 200, except for the gene FGF12. The g:Profiler web server was also used in analyzing the gene set of this subnetwork in terms of GO annotation and KEGG pathway. Results show that 7 of them are related to MAPK signaling pathway (p-value = 9.91e-10), that 7 of them are correlated to protein kinase binding (p-value = 2.57e-09), and that 6 of them are connected with MAPKKK cascade; furthermore, 3 of them are responsible for the MAP-kinase scaffold activity(p-value = 1.93e-10), which agree**s** well with the current knowledge of Diabetes [25], [26].

Our results for the three cases were also examined for further novel suggestions. We analyzed top-50 predictions in each case. We checked whether our predicted genes were already found to be involved in disease by searching for the online database or scientific publications. All of the published disease-gene associations that were not in our input data set were collected. There were 7 new associations for Breast Cancer (MIM:114480) in the recent OMIM database (January,2011) which ranked within top 50 in our result, but not in our data set. As for the Alzheimer Disease(MIM:104300), 5 novel genes within top 50 in our predicted results were verified in the online OMIM database. The gene LRP1 on chromosome 12 was studied with the Alzheimer Disease on 850 persons at the age of over 60 by Farrer et al. [27]. A 480-kb region encompassing the IDE gene was also investigated by Prince et al. [28] in relation to the Alzheimer Disease; furthermore, the cerebellar expression levels of IDE were measured by Zou et al. [29]. The same results were extracted for Diabetes Mellitus, Type 2, and 4 novel genes which were ranked among top 50 were verified in the online OMIM database.

We also computed the disease-gene association scores and corresponding ranks between all the 8919 genes characterized by the protein-protein interaction network and all the 5080 diseases in the disease similarity data set.

## Discussion

The success of our method can be attributed to a combination of several aspects. First, the large-scale disease similarity information is exploited. Second, which is more important, the disease similarity network and the protein-protein interaction network are coupled in a comprehensive and systematic way for the definition of the disease-gene association score function, and this is well in accord with the consistency between disease phenotypic overlap and genetic overlap. On one hand, the definition of disease-gene association score makes full use of the information implicated in both disease similarities and neighbouring genes comprehensively. On the other hand, not only the noise in the disease similarity information but also the self-loop in the protein-protein interaction network are considered in the computation of the disease-gene association scores. Third, an iterative algorithm was designed for the computation of the disease-gene association score matrix for all the diseases and all the candidate genes in the protein-protein interaction network.

Nevertheless, our method can be improved in the following ways. First, this method relies on the protein-protein interaction data which have a low coverage and a high false positive ratio, and the information on the isolated proteins in the network can not be exploited. In our protein-protein interaction data there are 57 isolated proteins which are known to be involved in some diseases. Second, the current disease similarity measurement is imprecise and subjective. It can be expected that this method would show more power if we could know more complete and reliable protein-protein interactions, together with a more standardized and objective disease description [30].

Two potential applications of our method should be noticed. First, the prioritization score for candidate genes can give some suggestions for further investigation. Second, the prioritization score can be exploited to identify disease-causing protein subnetworks, which are valuable for the study of the multi-factorial diseases, and this has been experienced successfully in PRINCE and this method.

## Methods

In this section, the principle of our method is illustrated first. Then the construction of several networks is defined and formulated. Finally, an iterative algorithm is designed for the computation of disease-gene association scores.

### Principle of the method

The observation that the genes implicated in the same or similar diseases lie close to each other in the protein-protein interaction network [1], [2], [3] has motivated the design of some computational approaches for prioritizing candidate genes involved in diseases. Our method is predicated on this simple observation together with the modular nature of the genetic diseases [3], [9] and the consistency between the phenotypic overlap [10] and genotypic overlap [11]. Here, when a candidate gene is prioritized for a disease, we consider the correlation of the two subnetworks separately induced by the neighbours of the gene in the protein-protein interaction network and the neighbours of the disease in the disease similarity network. That is, a single association between a gene and a disease is formulated iteratively by the correlation of the two subnetworks. This constraint can also be described as the fact that a gene is likely to be involved in a disease if the gene's neighbours are associated with the similar diseases. In our method, the association score between disease 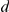 and gene 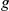 is formulated iteratively as the weighted sum of all the existing association scores between the neighbours of 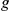 and the diseases similar to 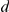. As in Figure 1, the association between gene 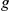 and disease 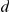 is measured over all the associations between 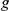 and 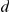
's similar diseases, the associations between 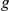
's neighbours and 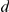, and the associations between 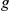
's neighbours and 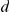
's similar diseases. In this figure, 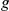
's neighbours are 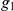, 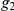, 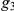, 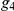, 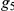, 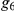, and 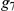, and 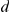
's similar diseases are 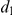, 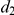, 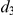, 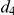, 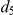, and 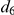. So, we compute the association score between 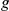 and 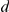 based on the known disease-gene associations iteratively as follows:
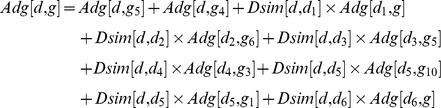



So are the associations between 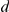 and 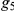, 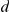 and 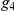, 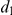 and 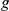, 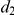 and 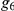, 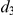 and 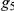, 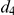 and 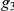, 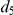 and 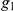, 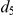 and 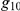, and 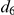 and 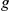 computed in the same iterative way.

We define a disease-gene association matrix in favor of computing and storing the association scores. The disease similarity network and the protein-protein interaction network are also constructed and incorporated into the formulation of the disease-gene association matrix in a simple and compact manner of matrix multiplication. As a result, an iterative algorithm is designed for the computation of the disease-gene association matrix. All this will be described in detail in the later part of this section.

### Network Construction

#### Filtered Disease similarity network

In our method, the disease similarity network is introduced, where the node in the network represents a disease, the edge connecting two nodes indicates that the two diseases are similar, and the weight of the edge indicates to what extent the two diseases are similar. We define a disease similarity matrix 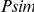 to model this network, in which 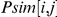 is the similarity score between disease 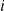 and disease 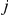. These disease similarities spanning 5080 diseases in the OMIM knowledge database were computed by van Driel et al. [10] by the text mining technique. In their analysis, similarity values in the range [0,0.3] were not informative, while the similarities in the range [0.6,1] showed significant functional similarity between corresponding diseases. So, in our method the parameter 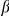 is used to filter out the disease similarities smaller than it, in order to purify the disease similarity network as much as possible.

#### Extended Protein-Protein interaction network

The protein-protein interaction network is modelled as matrix 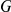, in which the value of 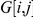 indicates whether the interaction between proteins 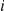 and 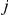 exists. The value “1” denotes that the interaction exists, and “0” denotes that the interaction does not exist. In our method, with regard to the association between disease 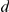 and gene 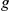, the associations between the diseases similar to 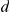 and the neighbours of 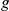, the associations between the diseases similar to 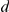 and the neigbours of 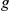, and the associations between 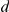 and the neighbours of 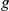 all need to be considered. So, we extend the protein-protein interaction network by adding the self-interactions of all the proteins into the interaction network. As a result, 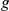 is a pseudo neighbour of 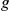 itself and will be counted when considering the neighbours of 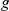, and this will be in favor of the iterative computation in a simple and compact manner of matrix multiplication. Here, the associations between 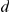 and neighbours of 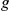 will be considered definitely because 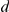 is the disease which is the most similar to itself, and that is true in the construction of the disease similarity network (all the elements on the diagonal being “1”).

#### Disease-Gene Association Network

We construct the disease-gene association network as the one where the node in the network can be either a disease or a gene and the weighted edge connecting a disease and a gene indicates to what extent the gene is involved in the disease. This network can also be regarded as a bipartite graph. In our method the disease-gene association network is expressed by a disease-gene association matrix 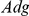, in which the element 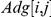 stores the association score of gene 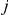 and disease 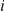 indicating the association strength between the gene and the disease. The matrix 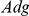 is initialized with the prior information on the disease-causing genes which are from the online OMIM database [12]. If the gene is known to be associated with the disease, the association score in the matrix is set to be “1”. With regard to the situation in which the disease 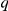 is not known to be associated with the gene 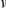, we deal with it in the way motivated by PRINCE [8]: the association score between 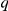 and 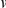 is defined as the similarity between the two diseases 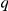 and 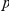. Here 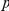 is chosen so carefully that 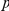 is not only the most similar to 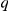 but also associated with the gene 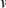 in our dataset. To eliminate the noise information brought about by disease similarities, the parameter 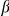 is also used to filter out the association score that is smaller than it.

### Algorithm

The input of our method includes both the protein-protein interaction network 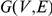, where 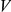 is the protein/gene set and 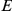 is the protein/gene interaction set (“protein” or “gene” will be used alternatively according to the context in the paper), and the disease similarity network 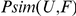, where 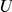 is the disease set and 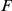 is the disease similarity set over every two diseases in 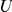. In our method, the disease-gene association matrix 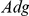 is defined over all the diseases in 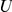 and all the genes in 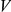 in Formula (2):
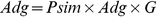
(2)


To solve the disease gene association matrix 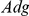 in Formula (2), we design an iterative algorithm. With regard to the prior information on the disease gene associations, the disease-gene association matrix is defined at the iteration 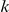 as Formula (3):
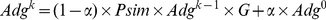
(3)


In Formula (3), the disease-gene association matrix is initialized as 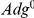 by the prior knowledge of the disease-gene associations. The parameter 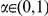 gives the relative importance between the constraints which are opposed by the assumption and the prior information. The constraint part of 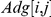 at the iteration 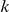 is defined as Formula (4):
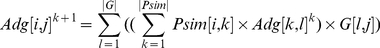
(4)where 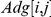 is computed based on the associations between 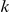 and 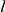, in which 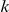 is over all the diseases similar to 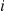 (including 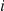) and 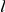 is over all the neighbours of 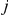 (including 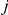). The iterative computation is similar in manner to that by PRINCE, and our method considers all the associations related to the association 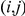 systematically and comprehensively while PRINCE considers just the information which will flow into the node 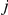 when querying the disease 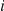 in one iteration.

The final score of each association is determined by the constraints opposed by both the protein-protein interaction network and the disease similarity network, and also by the prior knowledge. The iterative computation is controlled by the mean square deviation of the two neighbouring disease-gene association score matrixes. All the tests on the simulated data sets and the real data sets have shown that the iterative computation would converge eventually.

## References

[1] Gandhi TKB, Zhong J, Mathivanan S, Karthick L (2006). Analysis of the human protein interactome and comparison with yeast, worm and fly interaction datasets.. Nat Genet 2006.

[2] Oti M, Snel B, Huynen MA, Brunner HG (2006). Predicting disease genes using protein-protein interactions.. J Med Genet.

[3] Oti M, Brunner HG (2007). The modular nature of genetic diseases.. Clinical Genetics.

[4] Lage K, Karlberg EO, Storling ZM, Olason PI, Pedersen AG (2007). A human phenome-interactome network of protein complexes implicated in genetic disorders.. Nat Biotech.

[5] Kohler S, Bauer S, Horn D, Robinson PN (2008). Walking the interactome for prioritization of candidate disease genes.. American journal of human genetics.

[6] Wu X, Jiang R, Zhang MQ, Li S (2008). Network-based global inference of human disease genes.. Mol Syst Biol.

[7] Wu X, Liu Q, Jiang R (2009). Align human interactome with phenome to identify causative genes and networks underlying disease families.. Bioinformatics.

[8] Vanunu O, Magger O, Ruppin E, Sholomi T, Sharan R (2010). Associating genes and Protein Complexes with Disease via Network Propagation.. PloS Comput Biol.

[9] Goh KI, Cusick ME, Valle D, Childs B, Vidal M (2007). The human disease network.. PNAS.

[10] van Driel MA, Bruggeman J, Vriend G, Brunner HG, Leunissen JAM (2006). A text-mining analysis of the human phenome.. Eur J Hum Genet.

[11] Rzhetsky A, Wajngurt D, Park N, Zheng T (2007). Probing genetic overlap among complex human phenotypes.. Proc Natl Acad Sci USA.

[12] Hamosh A, Scott AF, Amberger JS, Bocchini CA, McKusick VA (2002). Online mendelian inheritance in man (omim), a knowledgebase of human genes and genetic disorders.. Nucl Acids Res.

[13] Cipher–Correlating protein interaction network and phenotype network to predict disease genes–website. [http://bioinfo.au.tsinghua.edu.cn/cipher/]. Accessed 2010 Aug. 20

[14] Peri S, Navarro JD, Kristiansen TZ, Amanchy R, Surendranath V (2004). Human protein reference database as a discovery resource for proteomics.. Nucleic Acids Res.

[15] MimMiner–A Online Mendelian Inheritance in Man Mining Tool–website.. http://www.cmbi.ru.nl/MimMiner/suppl.html.

[16] Anderson R, Chung F, Lang K (2006). Local graph partitioning using pagerank vectors..

[17] Jüri R, Meelis K, Hedi P, Jaanus H, Jaak V (2007). g:Profiler - a web-based toolset for functional profiling of gene lists from large-scale expriments.. Nuc Aci Res.

[18] g:Profiler–a web server for functional interpretation of gene lists–website.. http://biit.cs.ut.ee/gprofiler/index.cgi.

[19] Gaulton KJ, Mohlke KL, Vision TJ (2007). A computational system to select candidate genes for complex human traits.. Bioinformatics.

[20] Ashburner M, Ball CA, Blake JA, Botstein D, Butler H (2000). Gene Ontology: tool for the unification of biology.. Nature Genetics.

[21] Ogata H, Goto S, Sato F, Fujibuchi W, Bono H (1999). KEGG: Kyoto encyclopedia of genes and genomes.. Necleic Acids Research.

[22] Oldenburg RA, Meijers-Heijboer H, Cornelisse CJ, Devilee P (2007). Genetic susceptibility for breast cancer: how many more genes to be found?. Crit Rev Oncol Hemat.

[23] Schjeide BM, Schnack C, Lambert JC, Lill CM, Kirchheiner J, Tumani H (2011). The role of clusterin, complement receptor 1, and phosphatidylinositol binding clathrin assembly protein in Alzheimer disease risk and cerebrospinal fluid biomarker levels.. Arch Gen Psychiatry.

[24] Reitz C, Brayne C, Mayeux R, Medscape (2011). Epidemiology of Alzheimer disease.. Nat Rev Neurol.

[25] Huang YC, Lin JM, Lin HJ, Chen CC, Chen SY (2011). Genome-wide Association Study of Diabetic Retinopathy in a Taiwanese Population.. Ophthalmology.

[26] Liang H, Zhong Y, Huang Y, Chen G (2011). Type 1 receptor parathyroid hormone (PTH1R) influences breast cancer cell proliferation and apoptosis induced by high levels of glucose.. Med Oncol.

[27] Farrer LA, Bowirrat A, Friedland RP, Waraska K, Korczyn AD (2003). Identification of multiple loci for Alzheimer disease in a consanguineous Israeli-Arab community.. Hum Molec Genet.

[28] Prince JA, Feuk L, Gu HF, Johansson B, Gatz M (2003). Genetic variation in a haplotype block spanning IDE influences Alzheimer disease.. Hum Mutat.

[29] Zou F, Carrasquillo MM, Pankratz VS, Belbin O, Morgan K (2010). Gene expression levels as endophenotypes in genome-wide association studies of Alzheimer disease.. Neurology.

[30] Biesecker LG (2005). Mapping phenotypes to language: a proposal to organize and standardize the clinical descriptions of malformations.. Clin Genet.

